# Subacute neurological deficits and respiratory insufficiency due to intrathecal methotrexate

**Published:** 2021-11-29

**Authors:** Sjoerd I. P. J. de Faber, Pim G. N. J. Mutsaers, Martin J. van den Bent, Matthijs van der Meulen

**Affiliations:** ^1^Department of Neuro-Oncology, Erasmus MC Cancer Institute, Brain Tumor Center, University Medical Centre Rotterdam, Rotterdam, The Netherlands; ^2^Department of Neurology, Haga Hospital, The Hague, The Netherlands; ^3^Department of Hematology, Erasmus MC Cancer Institute, University Medical Center Rotterdam, Rotterdam, The Netherlands; ^4^Department of Neurology, Medisch Spectrum Twente, Enschede, The Netherlands

**Keywords:** methotrexate, leukoencephalopathy, neurotoxicity, acute lymphoblastic leukemia

## Abstract

**Background and Aim:**

We present a case of a 22-year-old male diagnosed with B-cell acute lymphoblastic leukemia who received intrathecal (IT) methotrexate (MTX) in addition to his systemic chemotherapy regime. During induction treatment, he presented with a rapidly progressive bilateral paresis, anarthria, and respiratory insufficiency requiring intubation. The brain magnetic resonance imaging showed bilateral lesions with diffusion restriction of the corona radiata/centrum semi-ovale without other abnormalities. He recovered spontaneously without neurological sequelae. The clinical course combined with the radiological findings is suspect for an IT-MTX-induced leukoencephalopathy.

**Relevance for Patients:**

Although neurological deficits after IT-MTX are rare and in most cases self-limiting, it should be recognized as a cause for rapid neurological decline after excluding other causes.

## 1. Case Description

**Figure 1:**
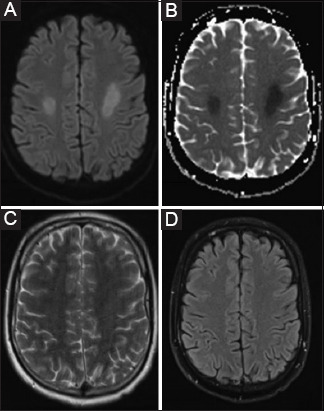
Magnetic resonance imaging cerebrum, 24 hours after the development of neurological deficits. (A) Diffusion-weighted imaging. (B) Apparent diffusion coefficient images: Bilateral diffusion restriction corona radiata/semi-oval center, (C) T2. (D) Fluid-attenuated inversion recovery: No abnormalities.

A 22-year-old male was diagnosed with precursor B-cell acute lymphoblastic leukemia (ALL) without cerebrospinal fluid (CSF) localization in December 2019. He was treated on a clinical trial combining first-line conventional induction chemotherapy (HOVON 146, EudraCT2017-000766-30) with blinatumomab. This regimen was combined with prophylactic intrathecal (IT) administrated methotrexate (MTX) (15 mg) and IT dexamethasone (4 mg). His first IT-MTX was given 1 week after his first systemic chemotherapy, in total, he received eight injections within 7 months. Two weeks after his 8^th^ IT-MTX, he was admitted to the hospital because of subacute neurological deterioration. Within 24 hours, he first developed a paralysis of his right arm in a few hours, followed by dysarthria and subsequently a paralysis of his left arm. Finally, he developed a facial diplegia and respiratory insufficiency for which he required mechanical ventilation. Computed tomography (CT), CT arteriography, and CT perfusion did not show any abnormalities. A magnetic resonance imaging (MRI) showed bilateral lesions with diffusion restriction in the centrum semi-ovale, no gadolinium contrast was administered (Figure 1).

**Figure 2:**
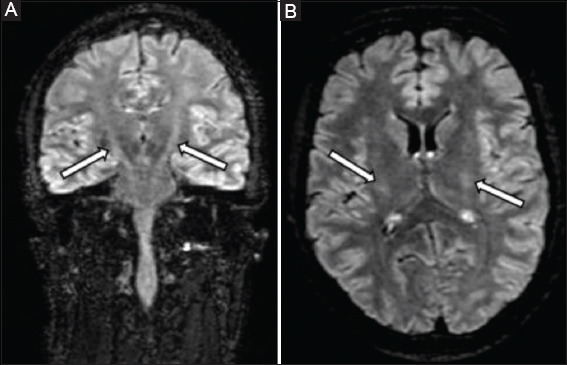
Magnetic resonance imaging cerebrum, at 4 months of follow-up. (A) T2. (B) Fluid-attenuated inversion recovery: Bilateral hyperintensities of the corticospinal tract (white arrows).

His condition improved spontaneously within several hours and the following day he did not need any ventilation support and he did not have any neurological sequelae. CSF analysis could not detect infections and no localization of ALL was found. Due to the spontaneous recovery and by excluding other causes, a diagnosis of MTX-induced neurotoxicity was made. Because of the MTX neurotoxicity, we decided to discontinue the IT prophylactic treatment. A follow-up MRI of the brain 4 months after recovery showed no signs of diffusion restriction but subtle bilateral T2 hyperintensities in the centrum semi-ovale continuing through the corticospinal tract until the pons (Figure 2). At physical examination, no neurological sequelae were found. The trial treatment was stopped and he switched to inotuzumab.

## 2. Discussion

The incidence of central nervous system (CNS) involvement in newly diagnosed adult ALL patients is <10%, but without prophylactic treatment, 50–75% of the patients will develop a CNS localization [1]. Over the years, multiple administrations of IT-MTX have become preferred over CNS irradiation for CNS prophylaxis in ALL [2]. IT-MTX is associated with the development of chemical meningitis and delayed leukoencephalopathy. It is important to administer IT corticosteroids together with MTX and to use 15 mg of folic acid, 24 hours after IT-MTX to replenish folic acid depletion [3]. Although rare, focal subacute neurotoxicity due to IT-MTX administration has been reported [4]. Clinically, patients can present with confusion, seizures, headache, and stroke like episodes, often developing 2–14 days after IT-MTX administration [5]. On MRI, diffusion restriction in the centrum semi-ovale or corona radiata, often bilateral, is reported in these cases although it has also been described in other regions such as thalamus, basal ganglia, cerebellum, and brainstem [5]. In general, patients recover rapidly from this toxicity with only symptomatic treatment. At follow-up imaging, areas of high intensity can be seen mainly in deep white matter on T2 and fluid-attenuated inversion recovery sequences which regress gradually between 1 and 7 months [6]. Our case is unusual, because of the severe paralysis leading to transient respiratory insufficiency.

## 3. Conclusion

Acute MTX-induced focal encephalopathy should be considered in patients who receive IT-MTX and present with (sub-) acute neurological deterioration.

### Conflicts of Interest

The authors declare no conflicts of interest.
